# Novel methods for reliability study of multi-dimensional non-linear dynamic systems

**DOI:** 10.1038/s41598-023-30704-x

**Published:** 2023-03-07

**Authors:** Oleg Gaidai, Jingxiang Xu, Ping Yan, Yihan Xing, Kelin Wang, Zirui Liu

**Affiliations:** 1 Shanghai Ocean University, Shanghai, China; 2 University of Stavanger, Stavanger, Norway

**Keywords:** Civil engineering, Statistics

## Abstract

This research presents two unique techniques for engineering system reliability analysis of multi-dimensional non-linear dynamic structures. First, the structural reliability technique works best for multi-dimensional structural responses that have been either numerically simulated or measured over a long enough length to produce an ergodic time series. Second, a novel extreme value prediction method that can be used in various engineering applications is proposed. In contrast to those currently used in engineering reliability methodologies, the novel method is easy to use, and even a limited amount of data can still be used to obtain robust system failure estimates. As demonstrated in this work, proposed methods also provide accurate confidence bands for system failure levels in the case of real-life measured structural response. Additionally, traditional reliability approaches that deal with time series do not have the benefit of being able to handle a system's high dimensionality and cross-correlation across several dimensions readily. Container ship that experiences significant deck panel pressures and high roll angles when travelling in bad weather was selected as the example for this study. The main concern for ship transportation is the potential loss of cargo owing to violent movements. Simulating such a situation is difficult since waves and ship motions are non-stationary and complicatedly non-linear. Extreme movements greatly enhance the role of nonlinearities, activating effects of second and higher order. Furthermore, laboratory testing may also be called into doubt due to the scale and the choice of the sea state. Therefore, data collected from actual ships during difficult weather journeys offer a unique perspective on the statistics of ship movements. This work aims to benchmark state-of-the-art methods, making it possible to extract necessary information about the extreme response from available on-board measured time histories. Both suggested methods can be used in combination, making them attractive and ready to use for engineers. Methods proposed in this paper open up possibilities to predict simply yet efficiently system failure probability for non-linear multi-dimensional dynamic structure.

## Introduction

Generally, it is quite challenging to calculate realistic structural system reliability by using conventional theoretical reliability methods^1–7^. The latter is often caused by many system freedom degrees and random variables that control dynamic systems. Theoretically, it is possible to estimate complicated structural systems' reliability straightforwardly by using sufficient data or direct Monte Carlo simulations^8–13^. The experimental or computational costs may be prohibitive for many complicated dynamic systems. Authors have developed a unique reliability approach for structural systems to lower measurement or computing costs as a result of the latter argument.

This work focuses on the general extreme value theory-based approach, in which it is anticipated that neither the physical dynamics of the water waves nor any other dynamic or environmental system would significantly contribute to the emergence of uncommon occurrences^14–20^. Although beyond the scope of our work, there have been some successful attempts to study extreme events in water waves (often called rogue or freak waves) with distributions that are uniquely determined by the dynamics of the physical system. For instance^20–24^, have shown that water waves departing from linear theory will modify their distribution from a Rayleigh type^25–30^, to a distribution dependent on the square root of the wave steepness. Similarly^31–35^, have shown that a Rayleigh distribution modified by a polynomial function of the ratio between height and water depth controls extreme events in Hurricane data. In addition, spectrum bandwidth seems to have different effects in extreme wave distribution depending on whether they are in deep waters^36–38^. Furthermore, ocean processes such as shoaling or wave-current systems that drive wave trains out of equilibrium have been experimental^39–42^ associated with increasing the occurrence of extreme waves by order of magnitude. However, it has been recently found that no established theoretical distribution to date, neither universal as Gumbel nor based on physical principles, can describe extreme wave statistics in a wide range of conditions^43–49^.

**Figure 1:**
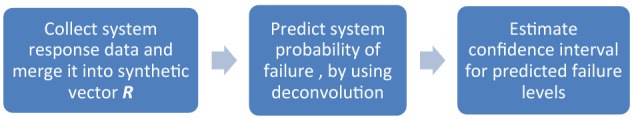
Flow chart for described methodology.

Figure 1 sketches the flow chart for the methodology suggested in this paper. For the definition of synthetic ${\varvec{R}}$ vector and more details see next Section. Note that methods introduced by authors here do not rely on Gumbel (or any other type) distribution type assumption. Instead, Gumbel-based extrapolation was used just for comparison. By applying the new approach to a collection of data from a real-world on-board measurement experiment aboard a container ship, this part aims to demonstrate the previously stated methodology's effectiveness. Ship dynamics is a well-known example of a highly non-linear, multi-dimensional, and cross-correlated dynamic system that is difficult to analyse. Furthermore, system reliability research is crucial for container ships traversing the Atlantic Ocean in actual, occasionally severe weather. Typically, it is considered that ocean waves constitute an ergodic random process (stationary and homogenous) within 3 h storm.

## Methods

Consider an offshore MDOF structure that is subjected to ergodic environmental loadings, such as those caused by the wind and waves in the area. The other option is to see a process dependent on external factors, whose fluctuation over time may be modelled as an independent ergodic process. Let ${X}_{1},\dots ,{X}_{{N}_{X}}$ be consequent in time local maxima of the process $X(t)$ at monotonously increasing discrete time instants ${t}_{1}^{X}<\dots <{t}_{{N}_{X}}^{X}$ in $(0,T)$. The analogous definition follows for other MDOF response components $Y\left(t\right), Z\left(t\right), \dots$ with ${Y}_{1},\dots ,{Y}_{{N}_{Y}};$ ${Z}_{1},\dots ,{Z}_{{N}_{Z}}$ and so on. For simplicity, all ${\varvec{R}}\left(t\right)$ components, and therefore its maxima are assumed to be non-negative. The aim is to estimate the system failure probability

1 $$1 - P = {\text{Prob}}\left( {X_{T}^{\max } > \eta_{X} \cup Y_{T}^{\max } > \eta_{Y} \cup Z_{T}^{\max } > \eta_{Z} \cup \ldots } \right)$$

with

2 $$\begin{aligned} P & = \mathop {\smallint \smallint \smallint }\limits_{{\left( {0,0,0, \ldots } \right)}}^{{\left( {\eta_{X} ,\eta_{Y} ,\eta_{Z} , \ldots } \right)}} p_{{X_{T}^{\max } ,Y_{T}^{\max } ,Z_{T}^{\max } , \ldots }} \left( {X_{T}^{\max } ,Y_{T}^{\max } ,Z_{T}^{\max } , \ldots } \right) \\ & \quad dX_{T}^{\max } dY_{{N_{Y} }}^{\max } dZ_{{N_{z} }}^{\max } \ldots \\ \end{aligned}$$

being the probability of non-exceedance for response components ${\eta }_{X}$, ${\eta }_{Y}$, ${\eta }_{Z}$,… critical values; $\cup$ denotes logical unity operation «or»; and ${p}_{{X}_{T}^{\mathrm{max}}, { Y}_{T}^{\mathrm{max}}, { Z}_{T}^{\mathrm{max}} , \dots }$ being joint probability density of the global maxima over the entire time span $(0,T)$.

In practice, however, it is not feasible to estimate the latter joint probability distribution directly ${p}_{{X}_{T}^{\mathrm{max}}, { Y}_{T}^{\mathrm{max}}, { Z}_{T}^{\mathrm{max}} , \dots }$ due to its high dimensionality and available data set limitations. In other words, the time instant when either $X\left(t\right)$ exceeds ${\eta }_{X}$, or $Y\left(t\right)$ exceeds ${\eta }_{Y}$, or $Z\left(t\right)$ exceeds ${\eta }_{Z}$, and so on, the system is regarded as immediately as failed. Fixed failure levels ${\eta }_{X}$, ${\eta }_{Y}$, ${\eta }_{Z}$,…are of course individual for each unidimensional response component of ${\varvec{R}}\left(t\right)$. ${X}_{{N}_{X}}^{\mathrm{max}}=\mathrm{max }\{{X}_{j}\hspace{0.17em};j=1,\dots ,{N}_{X}\}={X}_{T}^{\mathrm{max}}$, ${Y}_{{N}_{Y}}^{\mathrm{max}}=\mathrm{max }\{{Y}_{j}\hspace{0.17em};j=1,\dots ,{N}_{Y}\}={Y}_{T}^{\mathrm{max}}$,${Z}_{{N}_{z}}^{\mathrm{max}}=\mathrm{max }\{{Z}_{j}\hspace{0.17em};j=1,\dots ,{N}_{Z}\}={Z}_{T}^{\mathrm{max}}$, and so on.

Next, the local maxima time instants $\left[{t}_{1}^{X}<\dots <{t}_{{N}_{X}}^{X}; {t}_{1}^{Y}<\dots <{t}_{{N}_{Y}}^{Y}; {t}_{1}^{Z}<\dots <{t}_{{N}_{Z}}^{Z}\right]$ in monotonously non-decreasing order are sorted into one single merged time vector ${t}_{1}\le \dots \le {t}_{N}$.Note that ${t}_{N}=\mathrm{max }\{{t}_{{N}_{X}}^{X}, {t}_{{N}_{Y}}^{Y}, { t}_{{N}_{Z}}^{Z}, \dots \}$, $N={N}_{X}+{N}_{Y}+{ N}_{Z}+ \dots$. In this case ${t}_{j}$ represents local maxima of one of MDOF bio-system response components either $X\left(t\right)$ or $Y\left(t\right)$, or $Z\left(t\right)$ and so on. That means that having ${\varvec{R}}\left(t\right)$ time record, one needs continuously and simultaneously screen for unidimensional response component local maxima and record its exceedance of the MDOF limit vector $\left({\eta }_{X}, {\eta }_{Y}, {\eta }_{Z},...\right)$ in any of its components $X, Y, Z, \dots$. The local unidimensional response component maxima are merged into one temporal non-decreasing vector $\overrightarrow{R}=\left({R}_{1}, {R}_{2}, \dots ,{R}_{N}\right)$ in accordance with the merged time vector ${t}_{1}\le \dots \le {t}_{N}$. That is to say, each local maxima ${R}_{j}$ is the actual encountered local maxima corresponding to either $X\left(t\right)$ or $Y\left(t\right)$, or $Z\left(t\right)$ and so on. Finally, the unified limit vector $\left({\eta }_{1}, \dots ,{\eta }_{N}\right)$ is introduced with each component ${\eta }_{j}$ is either ${\eta }_{X}$, ${\eta }_{Y}$ or ${\eta }_{Z}$ and so on, depending on which of $X\left(t\right)$ or $Y\left(t\right)$, or $Z\left(t\right)$ etc., corresponding to the current local maxima with the running index $j$.

Next, a scaling parameter $0<\lambda \le 1$ is introduced to artificially simultaneously decrease limit values for all response components, namely the new MDOF limit vector $\left({\eta }_{X}^{\lambda },{ \eta }_{Y}^{\lambda }, {\eta }_{z}^{\lambda },...\right)$ with ${\eta }_{X}^{\lambda }\equiv \lambda {\cdot \eta }_{X}$, $\equiv \lambda {\cdot \eta }_{Y}$, ${\eta }_{z}^{\lambda }\equiv \lambda {\cdot \eta }_{Z}$, … is introduced. The unified limit vector $\left({\eta }_{1}^{\lambda }, \dots ,{\eta }_{N}^{\lambda }\right)$ is introduced with each component ${\eta }_{j}^{\lambda }$ is either ${\eta }_{X}^{\lambda }$, ${\eta }_{Y}^{\lambda }$ or ${\eta }_{z}^{\lambda }$ and so on. The latter automatically defines probability $P\left(\lambda \right)$ as a function of $\lambda$, note that $P\equiv P\left(1\right)$ from Eq. (1). Non-exceedance probability $P\left(\lambda \right)$ can be now estimated as follows:

3 $$\begin{aligned} P\left( \lambda \right) & = {\text{Prob}}\left\{ {R_{N} \le \eta_{N}^{\lambda } , \ldots ,R_{1} \le \eta_{1}^{\lambda } } \right\} \\ & = {\text{Prob}}\left\{ {R_{N} \le \eta_{N}^{\lambda }\,{|}\,R_{N - 1} \le \eta_{N - 1}^{\lambda } , \ldots ,R_{1} \le \eta_{1}^{\lambda } } \right\} \cdot {\text{Prob}}\left\{ {R_{N - 1} \le \eta_{N - 1}^{\lambda } , \ldots ,R_{1} \le \eta_{1}^{\lambda } } \right\} \\ & = \mathop \prod \limits_{j = 2}^{N} {\text{Prob}}\left\{ {R_{j} \le \eta_{j}^{\lambda }\,|\,R_{j - 1} \le \eta_{1j - }^{\lambda } , \ldots ,R_{1} \le \eta_{1}^{\lambda } } \right\} \cdot {\text{Prob}}\left( {R_{1} \le \eta_{1}^{\lambda } } \right) \\ \end{aligned}$$

In practice, the dependency between neighbouring ${R}_{j}$ is not always negligible; thus, the following one-step (called here conditioning level $k=1$) memory approximation is now introduced

4 $${\text{Prob}}\left\{ {R_{j} \le \eta_{j}^{\lambda }\,|\,R_{j - 1} \le \eta_{j - 1}^{\lambda } , \ldots ,R_{1} \le \eta_{1}^{\lambda } } \right\} \approx {\text{Prob}}\left\{ {R_{j} \le \eta_{j}^{\lambda }\,|\,R_{j - 1} \le \eta_{j - 1}^{\lambda } } \right\}$$

for $2\le j\le N$ (called here conditioning level $k=2$). The approximation introduced by Eq. (4) can be further expressed as

5 $${\text{Prob}}\left\{ {R_{j} \le \eta_{j}^{\lambda }\,|\,R_{j - 1} \le \eta_{j - 1}^{\lambda } , \ldots ,R_{1} \le \eta_{1}^{\lambda } } \right\} \approx {\text{Prob}}\left\{ {R_{j} \le \eta_{j}^{\lambda }\,|\,R_{j - 1} \le \eta_{j - 1}^{\lambda } , R_{j - 2} \le \eta_{j - 2}^{\lambda } } \right\}$$

where $3\le j\le N$ (will be called conditioning level $k=3$), and so on. The motivation is to monitor each independent failure that happened locally first in time, thus avoiding cascading local inter-correlated exceedances.

Equation (5) presents subsequent refinements of the statistical independence assumption. The latter type of approximation captures the statistical dependence effect between neighbouring maxima with increased accuracy. Since the original MDOF bio-process ${\varvec{R}}\left(t\right)$ was assumed ergodic and therefore stationary, the probability $p_{k} \left( \lambda \right) {:=} {\text{Prob}}\{R_{j} > \eta_{j}^{\lambda }\,{|}\,R_{j - 1} \le \eta_{j - 1}^{\lambda } ,\;R_{j - k + 1} \le \eta_{j - k + 1}^{\lambda } \}$ for $j\ge k$ will be independent of $j$ but only dependent on conditioning level $k$. Thus non-exceedance probability can be approximated as in the Naess–Gaidai method^43–49^, where

6 $$P_{k} \left( \lambda \right) \approx \exp \left( { - N \cdot p_{k} \left( \lambda \right)} \right),\quad k \ge 1$$

Note that Eq. (6) follows from Eq. (1) by neglecting $\mathrm{Prob}({R}_{1}\le {\eta }_{1}^{\lambda })\approx 1$, as the design failure probability is usually very small. Further, it is assumed *N* ″*k*.

Note that Eq. (5) is similar to the well-known mean up-crossing rate equation for the probability of exceedance^50,51^. There is obvious convergence with respect to the conditioning parameter $k$

7 $$P = \mathop {\lim }\limits_{k \to \infty } P_{k} \left( 1 \right);\quad p\left( \lambda \right) = \mathop {\lim }\limits_{k \to \infty } p_{k} \left( \lambda \right)$$

Note that Eq. (6) for $k=1$ turns into the quite well-known non-exceedance probability relationship with the mean up-crossing rate function

8 $$P\left( \lambda \right) \approx \exp \left( { - \nu^{ + } \left( \lambda \right)\,T} \right);\quad \nu^{ + } \left( \lambda \right) = \mathop \smallint \limits_{0}^{\infty } \zeta p_{{R\dot{R}}} \left( {\lambda ,\zeta } \right)d\zeta$$

where ${\nu }^{+}(\lambda )$ is the mean up-crossing rate of the response level $\lambda$ for the above assembled non-dimensional vector $R\left(t\right)$ assembled from scaled MDOF bio-system response $\left(\frac{X}{{\eta }_{X}}, \frac{Y}{{\eta }_{Y}}, \frac{Z}{{\eta }_{Z}}, \dots \right)$. In the above, the stationarity assumption has been used. The proposed methodology can also treat the non-stationary case. An illustration of how the methodology can be used to treat non-stationary cases is provided. Consider a scattered diagram of $m=1,..,M$ environmental states, each short-term bio-environmental state having a probability ${q}_{m}$, so that $\sum_{m=1}^{M}{q}_{m}=1$. The corresponding long-term equation is then

9 $$p_{k} \left( \lambda \right) \equiv \mathop \sum \limits_{m = 1}^{M} p_{k} \left( {\lambda ,m} \right)q_{m}$$

with ${p}_{k}(\lambda ,m)$ being the same function as in Eq. (7) but corresponding to a specific short-term environmental state with the number $m$. Next, by plotting ${\text{ln}}\left\{{\text{ln}}\left({p}_{k}(\lambda )\right)-{d}_{k}\right\}$ versus ${\text{ln}}\left({a}_{k}\lambda +{b}_{k}\right)$, often nearly perfectly linear tail behaviour is observed.

It is useful to do the optimisation on the logarithmic level by minimising the following error function *F* with respect to the four parameters ${a}_{k}, {b}_{k}, {c}_{k},{p}_{k},{q}_{k}$

10 $$F\left( {a_{k} ,b_{k} ,c_{k} ,p_{k} ,q_{k} } \right) = \mathop \smallint \limits_{{\lambda_{0} }}^{{\lambda_{1} }} \omega \left( \lambda \right)\left\{ {{\text{ln}}\left( {p_{k} \left( \lambda \right)} \right) - d_{k} + \left( {a_{k} \lambda + b_{k} } \right)^{{c_{k} }} } \right\}^{2} d\lambda ,\quad \lambda \ge \lambda_{0}$$

with ${\lambda }_{1}$ being a suitable distribution tail cut-off value, namely the largest wave height value, where the confidence interval width is still acceptable. Optimal values of the parameters ${a}_{k}, {b}_{k}, {c}_{k},{p}_{k},{q}_{k}$ may also be determined using a sequential quadratic programming (SQP) method incorporated in the NAG Numerical Library^52^. Weight function $\omega$ can be defined as $\omega \left(\lambda \right)={\left\{{\text{ln}}{\mathrm{CI}}^{+}\left(\lambda \right)-{\text{ln}}{\mathrm{CI}}^{-}\left(\lambda \right)\right\}}^{-2}$ with $\left({\mathrm{CI}}^{-}\left(\lambda \right),{ \mathrm{CI}}^{+}\left(\lambda \right)\right)$ being a confidence interval (CI), empirically estimated from the simulated or measured dataset^43–49^. When the parameter $c=\underset{k\to \infty }{\mathrm{lim}}{c}_{k}$ is equal to 1 or close to it, the distribution is close to the Gumbel distribution.

For any general ergodic wave height or wind speed process, the sequence of conditional exceedances over a threshold $\lambda$ can be assumed to constitute a Poisson process. However, in general, non-homogeneous one. Thus, for levels of $\lambda$ approaching $1$, the approximate limits of a *p-*% confidence interval (CI) of ${p}_{k}\left(\lambda \right)$ can be given as follows

11 $${\text{CI}}^{ \pm } \left( \lambda \right) = p_{k} \left( \lambda \right)\left( {1 \pm \frac{f\left( p \right)}{{\sqrt {\left( {N - k + 1} \right)p_{k} \left( \lambda \right)} }}} \right).$$

with $f(p)$ being estimated from the inverse normal distribution, for example, $f\left(90\%\right)=1.65$, $f\left(95\%\right)=1.96$. with $N$ being the total number of local maxima assembled in the analysed vector $\overrightarrow{R}$. Next, a novel extrapolation method is briefly introduced. Accurate extreme value prediction is a common and challenging engineering reliability task, especially when available data is scarce. Therefore, developing novel, efficient and accurate extrapolation techniques are of great practical importance. Let one consider a stationary stochastic process $X\left(t\right)$, either simulated or measured over a specific time span $0\le t\le T$, and which is represented as a sum of two independent stationary processes ${X}_{1}\left(t\right)$ and ${X}_{2}\left(t\right)$, namely

12 $$X\left( t \right) = X_{1} \left( t \right) + X_{2} \left( t \right)$$

Note that this paper aims at a general methodology applicable to extreme value predictions for a wide range of loads and responses for various vessels and offshore structures. For the process of interest $X\left(t\right)$ one may obtain marginal PDF (probability density function)${p}_{X}$ by two distinctive ways:

(A) By directly extracting ${p}_{X}^{A}$ from the available data set, i.e. time series $X\left(t\right)$,
(B) By separately extracting PDFs from the process components ${X}_{1}\left(t\right)$ and ${X}_{2}\left(t\right)$, namely ${p}_{{X}_{1}}$ and ${p}_{{X}_{2}}$, then applying convolution ${p}_{X}^{B}={\text{conv}}\left({ p}_{{X}_{1}},{ p}_{{X}_{2}}\right)$.

Both ${p}_{X}^{A}$ and ${p}_{X}^{B}$ are being approximations of the target PDF ${p}_{X}$. Approach (A) is more straightforward to use, however (B) would provide a more accurate estimate of the target PDF ${p}_{X}$. An advantage of using convolution in case (B) is based on the fact that convolution enables extrapolation of the directly extracted empirical PDF ${p}_{X}^{A}$, without pre-assuming any specific extrapolation functional class, e.g. generalised extreme value distributions (GEV) needed to extrapolate distribution tail towards design low probability level of interest. Note that most existing extrapolation methods, widely adopted in engineering practice, rely on assuming certain extrapolation functional classes, e.g.^43–49^. To name some of those most popular existing methods: Pareto based distribution peak over the threshold (POT)^50^.

The two independent component representation given by Eq. (12) is seldom available; therefore, one may look for artificial ways to estimate ${p}_{{X}_{1}}$ and ${p}_{{X}_{2}}$, or in the simplest case, find two identically distributed process components ${X}_{1}\left(t\right)$ and ${X}_{2}\left(t\right)$ with ${p}_{{X}_{1}}={ p}_{{X}_{2}}$. This paper is focused on the latter alternative, i.e. case when processes ${X}_{1}\left(t\right)$ and ${X}_{2}\left(t\right)$ are equally distributed. Therefore the current study goal would be, given directly estimated distribution ${p}_{X}$ as in option (A), to find component distribution ${p}_{{X}_{1}}$ such that

13 $$p_{X} = {\text{conv}}\left( { p_{{X_{1} }} , p_{{X_{1} }} } \right)$$

thus restricting this study only to a deconvolution case. In order to exemplify the latter idea regarding how to estimate the unknown distribution robustly ${p}_{{X}_{1}}$, and subsequently to improve (say extrapolate) the given empirical distribution ${p}_{X}.$

Accurate extreme value prediction becomes extremely challenging whenever there is a scarcity of data in engineering. Hence, a novel, efficient and accurate extrapolation method must be developed. Such a method better facilitates better design and reliability development.

In a case where the stationary stochastic process $X\left(t\right)$ which is either measured or simulated in a time span of $0\le t\le T$, and is then denoted as a sum of two independent stationary processes ${X}_{1}\left(t\right)$ and ${X}_{2}\left(t\right)$.

14 $$X\left( t \right) = X_{1} \left( t \right) + X_{2} \left( t \right)$$

Noteworthy with a general methodology, this paper hopes to better predict the extreme responses in engineering, with its primary focus on mechanical engineering, e.g. offshore jacket platform dynamics. Note that the method described here assumes a stationary dynamic system. In the case of non-stationary processes (long-term analysis), there will be a need to have stationary sub-parts in it (e.g. short-term analysis with 3-h sea states, taken from the scattered diagram).

The marginal PDF (probability density function)${p}_{X}$ can be acquired in two different ways for the process of interest $X\left(t\right)$:

(A) Using the available data set, i.e., time series $X\left(t\right)$, to directly extract ${p}_{X}^{A}$.
(B) Using the process components ${X}_{1}\left(t\right)$ and ${X}_{2}\left(t\right)$ to individually extract their PDFs from, namely ${p}_{{X}_{1}}$ and ${p}_{{X}_{2}}$, and then utilising convolution ${p}_{X}^{B}={\text{conv}}\left({ p}_{{X}_{1}},{ p}_{{X}_{2}}\right)$.

In both processes, ${p}_{X}^{A}$ and ${p}_{X}^{B}$ are the approximations of the target PDF ${p}_{X}$. The initial approach (A), is more straightforward; however, the second approach (B), gives a better and more accurate estimate of the target PDF ${p}_{X}$.

The convolution method in approach (B) is also advantageous since it facilitates the direct extraction of the empirical PDF ${p}_{X}^{A}$, without a presumption of any extrapolation functional class.

However, the two independent components from Eq. (10) are often unknown and thus ${p}_{{X}_{1}}$ and ${p}_{{X}_{2}}$ must be estimated. If not, in a more straightforward scenario, both the identically distributed process components ${X}_{1}\left(t\right)$ and ${X}_{2}\left(t\right)$ with ${p}_{{X}_{1}}={ p}_{{X}_{2}}$ must be estimated. The latter-mentioned method will be examined in this paper, considering that both the process of ${X}_{1}\left(t\right)$ and ${X}_{2}\left(t\right)$ are equally distributed. Thus, using the directly estimated distribution ${p}_{X}$ like approach A), the component distribution ${p}_{{X}_{1}}$ is derived

15 $$p_{X} = {\text{conv}}\left( { p_{{X_{1} }} , p_{{X_{1} }} } \right)$$

This limits this case to only a deconvolution scenario. The convolution of two vectors is characterised by the overlapping area of both the vectors, ${\varvec{u}}$ and ${\varvec{v}}$. Thus, convolution is algebraically similar to the multiplication of polynomials whose coefficients are the elements of ${\varvec{u}}$ and ${\varvec{v}}$. Let $m={\text{length}}\left({\varvec{u}}\right)$ and $n={\text{length}}\left({\varvec{v}}\right)$. Then ${\varvec{w}}$ is the vector of length $m+n-1$, whose $k$ th element is

16 $$w\left(k\right)=\sum_{j=1}^{m}u\left(j\right)v\left(k-j+1\right)$$

The sum is over all the values of $j$ that lead to legal subscripts for $u\left(j\right)$ and $v\left(k-j+1\right)$, specifically $j={\text{max}}\left(1,k+1-n\right):1:\mathrm{min}\left(k,m\right)$. When $m=n$, as will be the main case in this paper, the latter yields

17 $$\begin{aligned} & w\left( 1 \right) = u\left( 1 \right) \cdot v\left( 1 \right) \\ & w\left( 2 \right) = u\left( 1 \right) \cdot v\left( 2 \right) + u\left( 2 \right) \cdot v\left( 1 \right) \\ & w\left( 3 \right) = u\left( 1 \right) \cdot v\left( 3 \right) + u\left( 2 \right) \cdot v\left( 2 \right) + u\left( 3 \right) \cdot v\left( 1 \right) \\ & \cdots \\ & w\left( n \right) = u\left( 1 \right) \cdot v\left( n \right) + u\left( 2 \right) \cdot v\left( {n - 1} \right) + \cdots + u\left( n \right) \cdot v\left( 1 \right) \\ & \cdots \\ & w\left( {2n - 1} \right) = u\left( n \right) \cdot v\left( n \right) \\ \end{aligned}$$

From Eq. (13), ${\varvec{u}}={\varvec{v}}=\left(u\left(1\right),...,u\left(n\right)\right)$, reduced parts of ${\varvec{w}}$-components $w\left(n+1\right),...,w\left(2n-1\right)$ are obtained, when the index increases from $n+1$ to $2n-1$. The latter extends vector ${\varvec{w}}$ into the support domain, double the initial distribution support domain. In short, the distribution support length is doubled, $\left(2n-1\right)\cdot \Delta x\approx 2n\cdot \Delta x=2{X}_{L}$. When comparing with the initial distribution support length $n\cdot \Delta x={X}_{L}$. $\Delta x$, in this case, is the constant length of each discrete distribution bin. In short, the convolution convects the distribution tail properties further down the tail.

The representation of the empirical target distribution ${p}_{X}$ is ${\varvec{w}}=\left(w\left(1\right),...,w\left(n\right)\right)$ where $n$ is the length of distribution support $\left[0,{X}_{L}\right]$. In this paper, only the one-sided positive random variables, $X\ge 0$, are considered to minimise complexities. Moreover, only the deconvolution cases considered as Eq. (13) will be ${\varvec{u}}={\varvec{v}}$. Judging from Eq. (13), the vectors ${\varvec{w}}$ and ${\varvec{u}},$ has a corresponding distribution ${p}_{X}$ and ${p}_{{X}_{1}}$, respectively. The unknown components ${\varvec{u}}={\varvec{v}}=\left(u\left(1\right),...,u\left(n\right)\right)$, can be found from the given ${\varvec{w}}=\left(w\left(1\right),...,w\left(n\right)\right)$ in Eq. (13). It starts from the first component $u\left(1\right)=\sqrt{w\left(1\right)}$, then to the second component $u\left(2\right)=\frac{w\left(2\right)}{2u\left(1\right)}$, and until n component $u\left(n\right)$.

Through this method, a simple linear extrapolation of self-deconvoluted vector $\left(u\left(1\right),...,u\left(n\right)\right)$ towards $\left(u\left(n+1\right),...,u\left(2n-1\right)\right)$ is achieved. In short, ${p}_{{X}_{1}}$ has its tail extrapolated linearly within the following range $\left({X}_{L},2{X}_{L}\right).$ Thus now, the ${p}_{{X}_{1}}$ is known as a deconvoluted distribution and, in its discrete form, is characterised by a projected vector ${\varvec{u}}$. Based on Eq. (13), the vector ${\varvec{w}}$ is extended and extrapolated doubling the length of the initial distribution support domain. In short, the ${p}_{X}$ distribution support length is doubled, $\left(2n-1\right)\cdot \Delta x\approx 2n\cdot \Delta x=2{X}_{L}$, in comparison with the initial distribution support length $n\cdot \Delta x={X}_{L}$.

A smoothing tail procedure helps smooth the tails as the obtained measurements or Monte Carlo simulations are not smooth enough. Using the ${p}_{X}$ tail interpolation, the original distribution ${p}_{X}\left(x\right)$ tail has been introduced since a CDF distribution regularly has high tail values $x$. Furthermore, the Naess–Gaidai (NG) method was implemented since the tails become similar to ${\text{exp}}\left\{-{\left(ax+b\right)}^{c}+d\right\}$ with $a, b, c, d$ at $x\ge {x}_{0}$ Where $a, b, c, d$ are suitable constants. Similarly, the tail's linear extrapolation of ${p}_{{X}_{1}}$ is the preferred unbiased option. Other non-linear extrapolation approaches can be similarly used in the proposed method, but they typically introduce different assumptions and biases.

Distribution ${p}_{X}\left(x\right)\equiv { p}_{X}$ tail interpolation was performed, as CDF distribution tail is generally quite regular for high tail values $x$. At the same time, other non-linear extrapolation approaches can easily plug into the proposed method, but then certain assumptions and biases would be introduced. The NG extrapolation method has been used; see “Introduction” section.

In the following, numerical results are presented based on the proposed deconvolution extrapolation method outlined in the previous Section. As discussed in the previous Section, the deconvolution extrapolation technique does not pre-assume any specific extrapolation functional class needed to extrapolate the distribution tail.

Since in most reliability analysis engineering applications, it is more important to estimate the probability of exceedance, i.e. 1-CDF where CDF stands for cumulative density function, rather than the marginal PDF, subsequently in this paper notation ${f}_{X}$ will stand for the probability of exceedance 1-CDF, analogous to the marginal probability density function PDF ${p}_{X}$ in the previous section. However, the proposed methodology may be suitable for any sufficiently regular monotonously decreasing either concave or convex function tail.

To validate the above-suggested extrapolation methodology, the «shorter» version of the original data set has been used for extrapolation for the sake of comparison with predictions based on the entire «longer» data set. Therefore, this work aims to demonstrate that the recommended extrapolation approach is at least a few orders of magnitude efficient.

The description above shows that an iterative technique may be used, whereas a marginal PDF can be created using 1-CDF and then generate a new artificial smoother CDF using integration. The latter can significantly facilitate extrapolation if there are distribution tail irregularities due to the scarcity of the underlying data set.

Next, the procedure of discrete convolution, or rather de-convolution (as the purpose was to find a deconvoluted 1-CDF distribution ${f}_{{X}_{1}}$, given the empirical distribution ${f}_{X}$) outlined in the previous section, is based on sequential solving of Eq. (4). Since the resulting deconvoluted values ${\varvec{u}}=\left(u\left(1\right),...,u\left(n\right)\right)$ are typically following a monotonously decreasing pattern (the same was assumed for the empirical parent distribution ${f}_{X}$), it appears that some last values of resulting vector ${\varvec{u}}$, say $\left(u\left(n-L\right),\dots ,u\left(n\right)\right)$ for some $L<n$ may become negative. Because positive numbers may only represent distributions, the latter is a numerical mistake and cannot be accepted. The following scaling method has been proposed to address that numerical difficulty. The lowest positive value ${f}_{L}$ of the given distribution tail of ${f}_{X}$ is taken as a pivot value. The scaling then is simply a linear transformation along the vertical *y*-axis of the distribution on the decimal logarithmic scale

18 $$g_{X} = { }\mu \left( {{\text{log}}_{{{10}}} \left( { f_{X} } \right) - {\text{log}}_{{{10}}} \left( { f_{L} } \right)} \right) + {\text{log}}_{{{10}}} \left( { f_{L} } \right)$$

with ${g}_{X}\left(x\right)$ being scaled ${\text{log}}_{10}$ version of the empirical base distribution ${f}_{X}$, with the reference level ${f}_{L}$ being intact. The scaling coefficient $\mu$ is conveniently chosen to avoid the occurrence of negative components in the resulting ${f}_{{X}_{1}}$. For both numerical examples studied in this paper, $\mu =1/3$ served that purpose well. Then when ${f}_{{X}_{1}}$ was found, and back convolution ${\widetilde{f}}_{X}={\text{conv}}\left({ f}_{{X}_{1}},{ f}_{{X}_{1}}\right)$ as in Eq. (13) was done, the inverse scaling with ${\mu }^{-1}$ was performed to restore the original scale, with ${\widetilde{f}}_{X}$ being extrapolated version of ${f}_{X}$.

## Results

A Post-Panamax cargo ship named MSC Napoli capsized in January 2007. MSC Napoli broke in two places: amidships, against a pillar bulkhead and in the engine room. Another Post-Panamax cargo ship, MOL Comfort, broke in June 2013^53,54^. Even though these two ships may not have been constructed and authorised in accordance with best practices, giving them less collapse strength than other ships of comparable sizes, both ships capsized owing to the overloading of the hull girders. Such catastrophic events require extensive investigations as they shock the business, particularly the container ship sector, in these two incidents^39–42^.

This section shows how the technique mentioned above is used in practice. Motion sensors were installed on the 2800TEU Panamax container ship during its transatlantic trips. The mid and aft ship panel stresses and the ship roll angle were selected as components *X*, *Y*, and *Z* to form an example of a three-dimensional (3D) dynamic system.

Unidimensional extreme response values were chosen as crucial thresholds, which led to vascular failure. These values generally equate to a 25-year return time. Namely ${\eta }_{X}=140$ MPa, ${\eta }_{Y}=110$ MPa, ${\eta }_{Z}=$ 28 degrees; see^45–49^.

**Figure 2:**
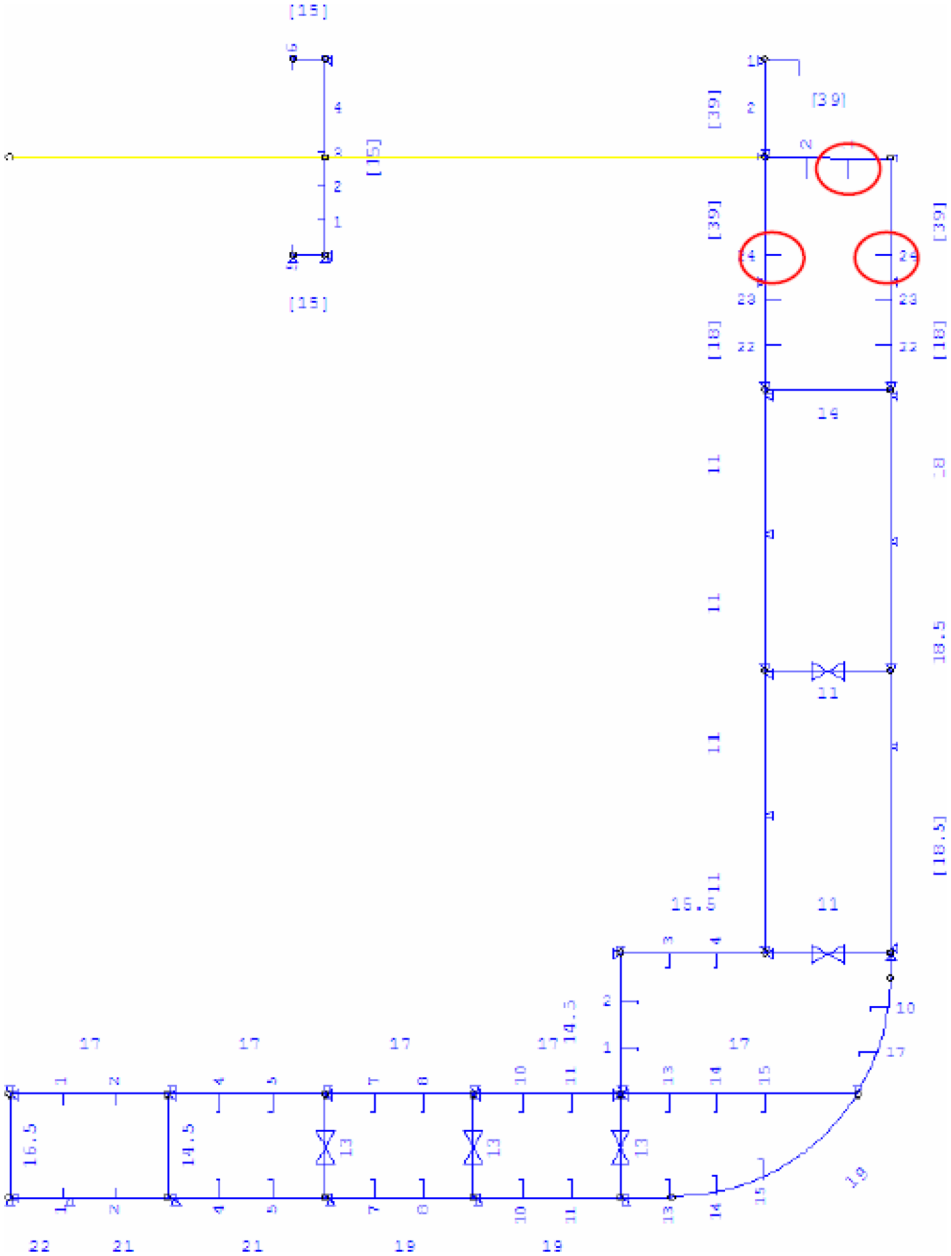
Layout of the mid-ship cross-section with measurement position in the upper deck and crack positions.

Compared to contemporary Post-Panamax container ships, this specific Panamax container ship from the late 1990s has a small bow flare angle. Hence the amount of whipping on this ship will be quite minor. Routing, a human aspect, will control the excessive response. Figure 2 presents TEU2800 mid-ship on-board strain sensors placement along with observed crack positions. Similarly, sensors were placed aft of the vessel, resulting in measured stresses in the longitudinal direction on a flat bar below the upper deck. Sensor placement was done according to DNV container vessel rules and regulations^55–59^. In order to unify all three measured time series $X, Y, Z$, the following scaling was performed

19 $$X \to \frac{X}{{\eta_{X} }},\quad Y \to \frac{Y}{{\eta_{Y} }},\quad Z \to \frac{Z}{{\eta_{Z} }}$$

making all three responses non-dimensional and having the same failure limit equal to 1. Next, all local maxima from three measured time series were merged into one single time series by keeping them in time non-decreasing order: $\overrightarrow{R}=\left(\mathrm{max}\left\{{X}_{1},{Y}_{1},{Z}_{1}\right\},\dots ,\mathrm{max}\left\{{X}_{N},{Y}_{N},{Z}_{N}\right\}\right)$ with each set $\mathrm{max}\left\{{X}_{j},{Y}_{j},{Z}_{j}\right\}$ being sorted according to temporally non-decreasing occurrence times of these local maxima. Figure 4 left presents an example of a non-dimensional assembled vector $\overrightarrow{R}$, consisting of assembled local maxima of TEU2800 mid and aft stresses along with vessel roll angle; $\lambda >0.2$ cut-on limit was used for illustrative purposes, as lower values $\lambda \ge 0$ are obviously are not relevant for the failure probability distribution tail extrapolation towards the target $\lambda =1$. Note that vector $\overrightarrow{R}$ does not have a physical meaning on its own, as it was assembled of different response components with different units of measurement (MPa and angular degrees in this case). Index $j$ is just a running index of local maxima encountered in a non-decreasing time sequence. The «shorter» data record has been generated by taking each tenth data point from the «longer» deck panel stress data record. Therefore, the «shorter» data record had an equivalent time length of only one year.

**Figure 3:**
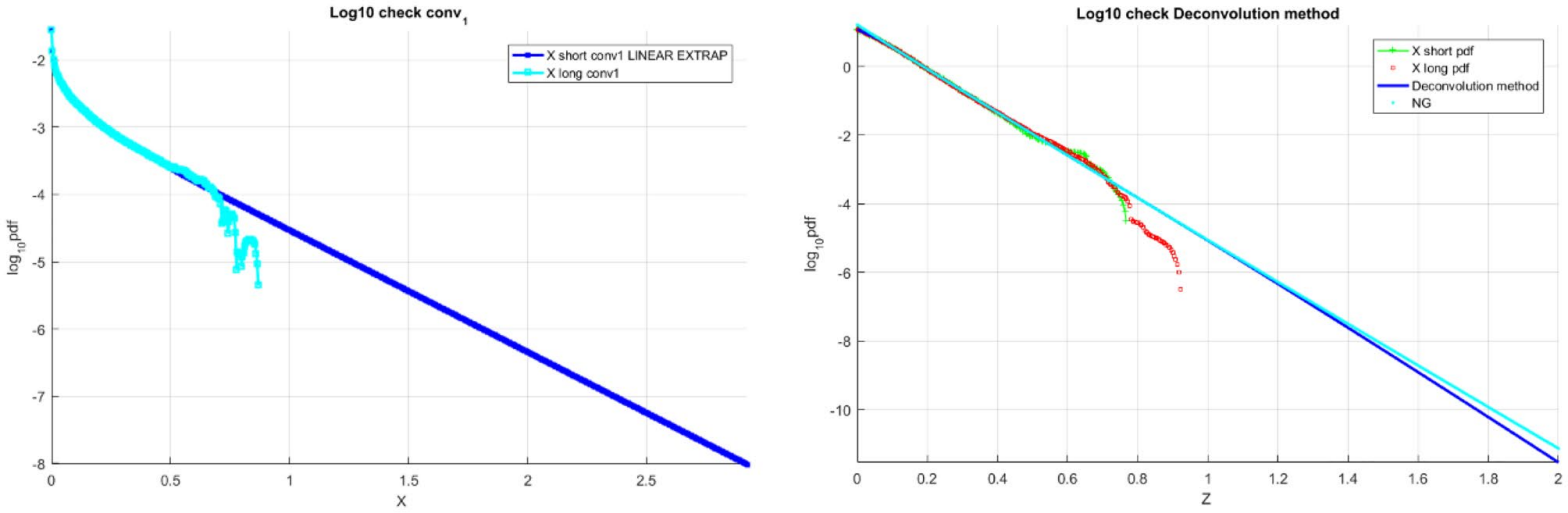
TEU vessel data. Left: scaled ${f}_{{X}_{1}}$ tail on the decimal log scale for the «shorter» data (cyan), linearly extrapolated (dark blue). Right: unscaled raw «shorter» data (green) ${f}_{X}$ tail on the decimal log scale, extrapolated by the deconvolution method (dark blue), along with «longer» raw data (red) and NG extrapolation (cyan).

Figure 3 on the left presents the «shorter» data record ${f}_{{X}_{1}}$ tail, obtained by deconvolution as in Eq. (13), and subsequently linearly extrapolated in the terminal tail section to cover the ${X}_{1}$ range matching the «longer» data record. Figure 3 on the right presents the final unscaled results of the proposed in this paper technique, namely the «shorter» decimal log scale ${f}_{X}$ tail, extrapolated by deconvolution, along with «longer» data distribution tail and NG extrapolation.

It is seen from Fig. 3 on the right that the proposed method performs quite well, being based on the «shorter» data set and delivering distribution quite close to the one based on the «longer» data set.

**Figure 4:**
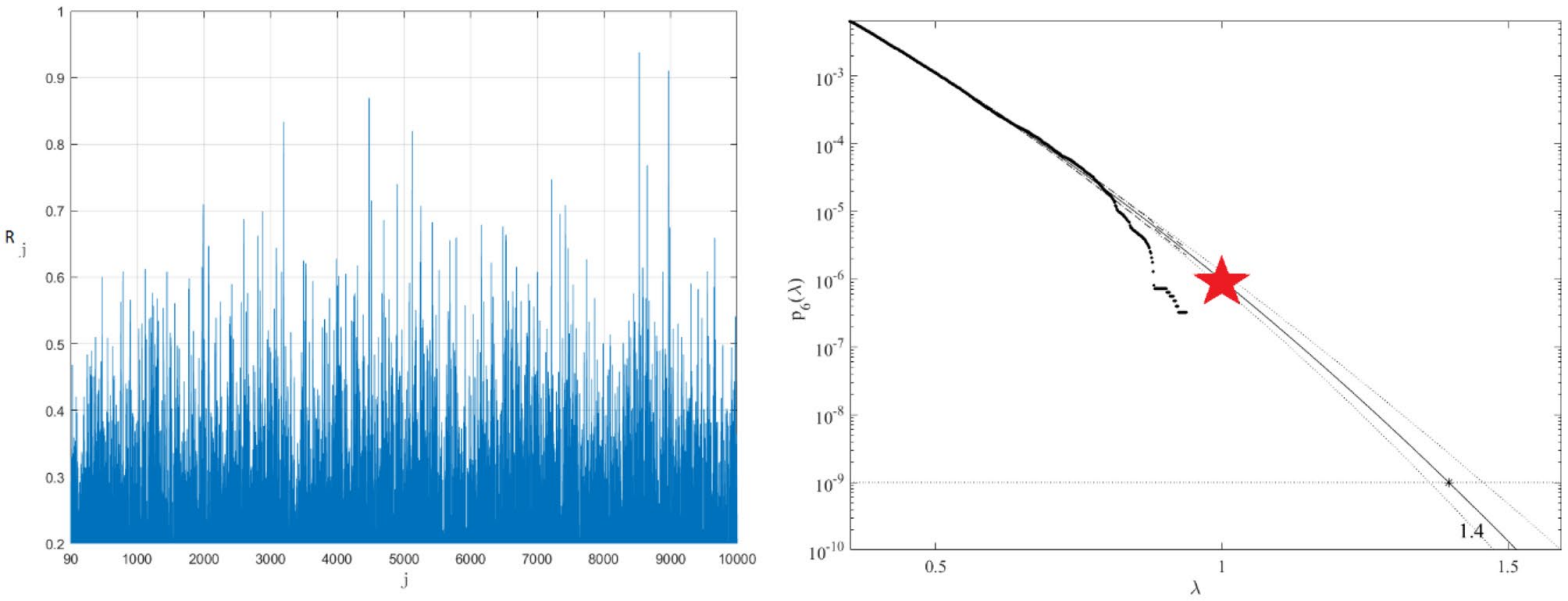
Left: Example of non-dimensional assembled 3D vector $\overrightarrow{R}$. Right: Extrapolation of ${p}_{k}\left(\lambda \right)$ towards critical level (indicated by a star) and beyond,$k=6$. Extrapolated 95% CI indicated by dotted lines.

Figure 4 right presents extrapolation according to Eq. (9) towards failure state with 25 year return period, which is 1, and somewhat beyond, $\lambda =0.4$ cut-on value was used. Dotted lines indicate extrapolated 95% confidence interval according to Eq. (10). According to Eq. (5) $p\left(\lambda \right)$ is directly related to the target failure probability $1-P$ from Eq. (1). Therefore, in agreement with Eq. (5) system failure probability $1-P\approx {1-P}_{k}\left(1\right)$ can be estimated. Note that in Eq. (5) $N$ corresponds to the total number of local maxima in the unified response vector $\overrightarrow{R}$. Conditioning parameter $k=6$ was found to be sufficient, due to convergence occurrence with respect to $k$, see Eq. (6). Figure 4 exhibits quite narrow 95% CI; the latter is due to a substantial amount of data used in this study namely over 70 trans-Atlantic voyages of the same vessel.

While being novel, the methodology described above has a clear advantage of utilising available measured data set quite efficiently, due to its ability to treat system multi-dimensionality and perform accurate extrapolation based on a relatively limited data set.

## Conclusions

Traditional reliability techniques with time series do not have the benefit of effectively dealing with highly dimensional systems and cross-correlation between various system responses. The methodology's primary asset is the capacity to analyse the reliability of high dimensional non-linear dynamic systems.

This study examined ship dynamic reaction time series collected aboard a TEU2800 Panamax container ship over more than 70 transatlantic journeys between 2007 and 2010. The vessel reliability as a multi-dimensional system used novel reliability methodologies in real-time. The suggested method's theoretical justification is explained in depth. It should be noted that while using direct measurement or Monte Carlo simulation to analyse the reliability of dynamic systems is appealing, the complexity and high dimensionality of dynamic systems necessitate the development of novel, accurate, and robust techniques that can handle the available data while utilising it as effectively as possible.

The approaches discussed in this study have already been shown effective when applied to a wide variety of simulation models, but only for one-dimensional system responses. In general, highly precise predictions were made. This work focused on a general-purpose, reliable, and user-friendly multi-dimensional reliability approach. The proposed strategy yielded an excellent confidence interval, as demonstrated. As a result, the recommended technique might be helpful in a range of non-linear dynamic systems reliability investigations. Time series responses can be measured and numerically simulated and studied.

In contrast to prior reliability techniques, the new technique does not call for restarting Monte Carlo-type numerical simulation every time the system fails. As this paper's illustration of measured structural reaction shows, it is also feasible to accurately anticipate the likelihood of system collapse.

To sum up, the recommended technique may be used in various engineering fields. By no means does the given naval architecture example restrict the potential applications of a new methodology.

## Data Availability

The generated data can be accessed upon reasonable request from the corresponding author Dr. Jingxiang Xu, jxxu@shou.edu.cn.
